# Severe Retinal Whitening in an Adult with Cerebral Malaria

**DOI:** 10.4269/ajtmh.2009.08-0663

**Published:** 2009-06

**Authors:** Richard J. Maude, Mahtab Uddin Hassan, Nicholas A. V. Beare

**Affiliations:** Faculty of Tropical Medicine, Mahidol University, Bangkok, Thailand; Centre for Clinical Vaccinology and Tropical Medicine, Nuffield Department of Clinical Medicine, John Radcliffe Hospital, Oxford, United Kingdom; Chittagong Medical College, Chittagong, Bangladesh; St. Paul's Eye Unit, Royal Liverpool University Hospital, Liverpool, United Kingdom

**Figure 1.**
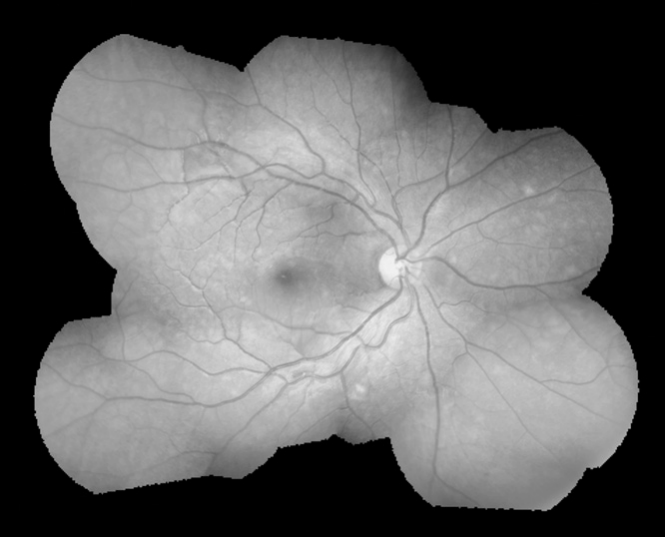
Composite digital photograph of the right retina of a 24-year-old man with cerebral malaria and severe malarial retinopathy. This figure appears in color at www.ajtmh.org.

A composite digital photograph of the right retina of a 24-year-old Bangladeshi man with non-fatal cerebral malaria was taken on admission to hospital (Figure 1). He presented with 1 day of unconsciousness, pulmonary edema, and blackwater fever. Peripheral blood parasitemia was 79 *Plasmodium falciparum* per 1,000 red cells and hemoglobin was 10.9 g/dL. He recovered consciousness within 48 hours of starting intravenous artesunate and was discharged home after 6 days. Visual function and neurologic examination were normal on discharge.

The image is comprised of 14 separate photographs taken with a Genesis D (Kowa, Japan) handheld retinal camera. It shows severe malarial retinopathy with small patches of retinal whitening involving the entire macula and extensive areas of the peripheral retina, in particular temporal to the central fovea and nasal to the optic nerve head (some glare is present along the temporal vascular arcades). Malarial retinal whitening has a distribution that is unique to malaria^1^ and is present in 50% of children with cerebral malaria. 1 In adults, it has previously only been described in two Malawians with cerebral malaria, both of whom had mild changes. 2

The handheld retinal camera can be used at the bedside and, unlike conventional retinal photography with a table-top camera, does not require a cooperative, sitting patient.
